# Use of intravenous sodium bicarbonate in neonatal intensive care units in Italy: a nationwide survey

**DOI:** 10.1186/s13052-021-00955-3

**Published:** 2021-03-11

**Authors:** Luca Massenzi, Roberto Aufieri, Silvia Donno, Rocco Agostino, Andrea Dotta, on behalf of the Neonatal Pharmacotherapy Study Group of the Italian Society of Neonatology (SIN)

**Affiliations:** 1 Department of Pediatrics and Neonatology, “S. Giovanni Calibita” Fatebenefratelli Hospital, Via di Ponte Quattro Capi 39, 00186 Rome, Italy; 2 Division of Neonatology and Neonatal Intensive Care Unit, ASL Roma 2 – Ospedale Sant’Eugenio, Rome, Italy; 3 INVALSI – Istituto nazionale per la valutazione del sistema educativo di istruzione e formazione, Rome, Italy; 4 Division of Neonatology, Villa Margherita Private Nursing Home, Rome, Italy; 5 Neonatal Intensive Care Unit, Department of Neonatology, Bambino Gesù Children’s Hospital, Rome, Italy

**Keywords:** Infant, newborn, Sodium bicarbonate, Acid-Base imbalance, Surveys and questionnaires, Drug therapy

## Abstract

**Background:**

Metabolic Acidosis (MA) is a disturbance of the acid-base balance that can occur in preterm and critically ill term neonates due to different etiologies. Intravenous sodium bicarbonate (SB) has been traditionally used to correct such unbalance, despite the lack of evidence about its safety and efficacy. In literature, reported undesirable effects of treatment with SB in neonates include worsening of intracellular acidosis, impairment of myocardial function, cerebral blood flow fluctuations and intracranial hemorrhage. A national survey was conducted by the Neonatal Pharmacotherapy Study Group of the Italian Society of Neonatology with the aim to assess and describe attitudes and practices concerning the use of SB, particularly for the treatment of MA in Italian NICUs.

**Methods:**

A questionnaire regarding treatment of MA and SB prescription habits was sent to the directors of 120 Italian NICUs from June 2017 to March 2018.

**Results:**

The survey response rate was 97.5% (117/120 centers). Findings showed that in 55% of the surveyed NICUs (64/117 units) it is common practice to correct MA with intravenous SB. On the other hand, the remaining 45% of the units try to solve the metabolic disturbances adopting different approaches (improving perfusion, adjusting ventilation parameters or increasing blood volume). Moreover, to prevent the occurrence of MA, 37.6% of the NICUs (44/117) include buffer salts (lactate, acetate or both) in parenteral nutrition prescriptions. SB is also used as a treatment for other conditions, mainly pathologies with bicarbonate loss and tubular acidosis (respectively in 53.8 and 32.5% of the NICUs).

**Conclusion:**

This survey showed how SB is a commonly used treatment for MA in more than half of Italian NICUs, with indications and prescription criteria that significantly vary across centers. Based on current knowledge, it is reasonable to suggest that the management of neonatal MA should be firstly directed to identify the underlying disorders. Thus, the use of SB should be reserved only for selected cases, also considering the severity of SB adverse effects and the lack of evidence about its efficacy. Guidance for the management of MA is required to harmonize practices and reduce the use of potentially inappropriate and unsafe treatments.

**Supplementary Information:**

The online version contains supplementary material available at 10.1186/s13052-021-00955-3.

## Background

Despite being a common and largely debated issue, the management of neonatal metabolic acidosis (MA) still represents a challenge for clinicians, mainly due to the lack of evidence suggesting the most suitable and effective treatment. Therapeutic uncertainty can be mostly attributed to the different pathogenic pathways at the origin of MA: immaturity of the renal system, perinatal asphyxia, hypovolemia, sepsis, congenital heart diseases, renal or gastrointestinal losses and inborn errors of the metabolism, among others [1, 2]. The historically established empiric correction of MA with intravenous sodium bicarbonate (SB) is a controversial and largely debated practice. Indeed, it is understandable that one single drug may not be effective for such a large number of different etiologies. Moreover, current evidence suggests that intravenous SB could represent a harmful treatment for patients with MA. Although numerous reports showed significant negative effects due to SB infusions, such as fluctuations in cerebral and cardiovascular hemodynamic [3, 4], increased rates of severe intraventricular hemorrhage (IVH) and mortality [5], SB is still used for treating MA in many neonatal intensive care units, as reported in a European survey conducted by Saenz P. et al. in 2011. The authors themselves commented that their study indicated the presence of a “*gap between scientific evidence and clinical practice”* [6].

The aim of this study was to investigate the attitude and practices related to the use of intravenous SB with particular attention to the treatment of MA in Italian NICUs. For this purpose, in 2017, a national survey was carried out by the Neonatal Pharmacotherapy study group of the Italian Society of Neonatology (SIN).

## Methods

We conducted a questionnaire-based study across Italian NICUs from June 2017 to March 2018.

### Questionnaire

A 14-item questionnaire was developed by the Neonatal Pharmacology Study Group of the SIN. The questionnaire inquired about: NICU characteristics (volume of activity); treatment of MA (use of intravenous SB or alternative therapeutic strategies); SB dosage, preparation and administration; presence of buffer systems in the parenteral nutrition (PN); use of intravenous SB for other conditions. All questions were loaded on Google Forms Website (Google Inc., CA/USA), a free tool for creating online survey forms. The survey form was pre-tested for clarity purposes by other members of the study group. The questionnaire required about 15 min to be filled in and sent.

### Web-based survey

Email addresses of the directors of the Italian NICUs were initially collected from the database of the SIN administrative office, after receiving consent for data utilization from the board of the society. In June 2017 a cover letter was sent to the directors of the NICUs requesting to complete and submit the online questionnaire available at the hyperlink https://www.goo.gl/forms/nMn3KTyNb6xNQv9K2 (File S1). In September 2017, considering that only 49 units had submitted the completed questionnaire, a review of the NICU directors’ contact list was carried out by LM and RA, and a new letter was sent to all directors, including those whose contacts resulted as being previously missing, incorrect or obsolete (File S2). In January 2018, all non-responders received a reminder email. Those who did not reply were finally contacted by phone and encouraged to participate in the survey. Data collection ended in March 2018. Participation was voluntary, and no financial rewards, nor other incentives were offered for the survey. The identity of each NICU director was kept confidential throughout the data collection and analysis.

### Statistical analysis

By means of Google Forms (Google Inc., CA/USA), the questionnaires were converted into an Excel file (Microsoft, Seattle, WA). Subsequently, the questionnaires and the automatically generated file were re-checked for possible inconsistencies before inclusion in the database to be analyzed.

Statistical analysis consisted of descriptive statistics: continuous variables are presented as mean values ± standard deviation (SD); categorical variables are presented as absolute and relative frequencies (%). Data are provided as absolute numbers and relative frequencies (%). The χ^2^ test or Fisher’s exact test was used to compare differences in categorical variables among groups. A *p* values less than 0.05 was considered statistically significant, and *p*-values were based on two-tailed tests. Statistical analysis was performed using SPSS for Windows (SPSS, Inc., Chicago, IL).

## Results

The overall survey response rate was 97.5% (117/120 contacted units). All the 117 filled in questionnaires were included in the analysis.

### NICU features

Of the 117 investigated NICUs, the majority (83.8%) reside in perinatal centers that take care of more than 1000 neonates per year, 39% with 1000–2000 births/year and 45% with more than 2000 births/year. Four NICUs (3.4%) hospitalize only out-born patients; meanwhile, the remaining 12.8% (15/117) is active with less than 1000 births/year.

### Use of intravenous SB for treatment of MA

**Fig. 1:**
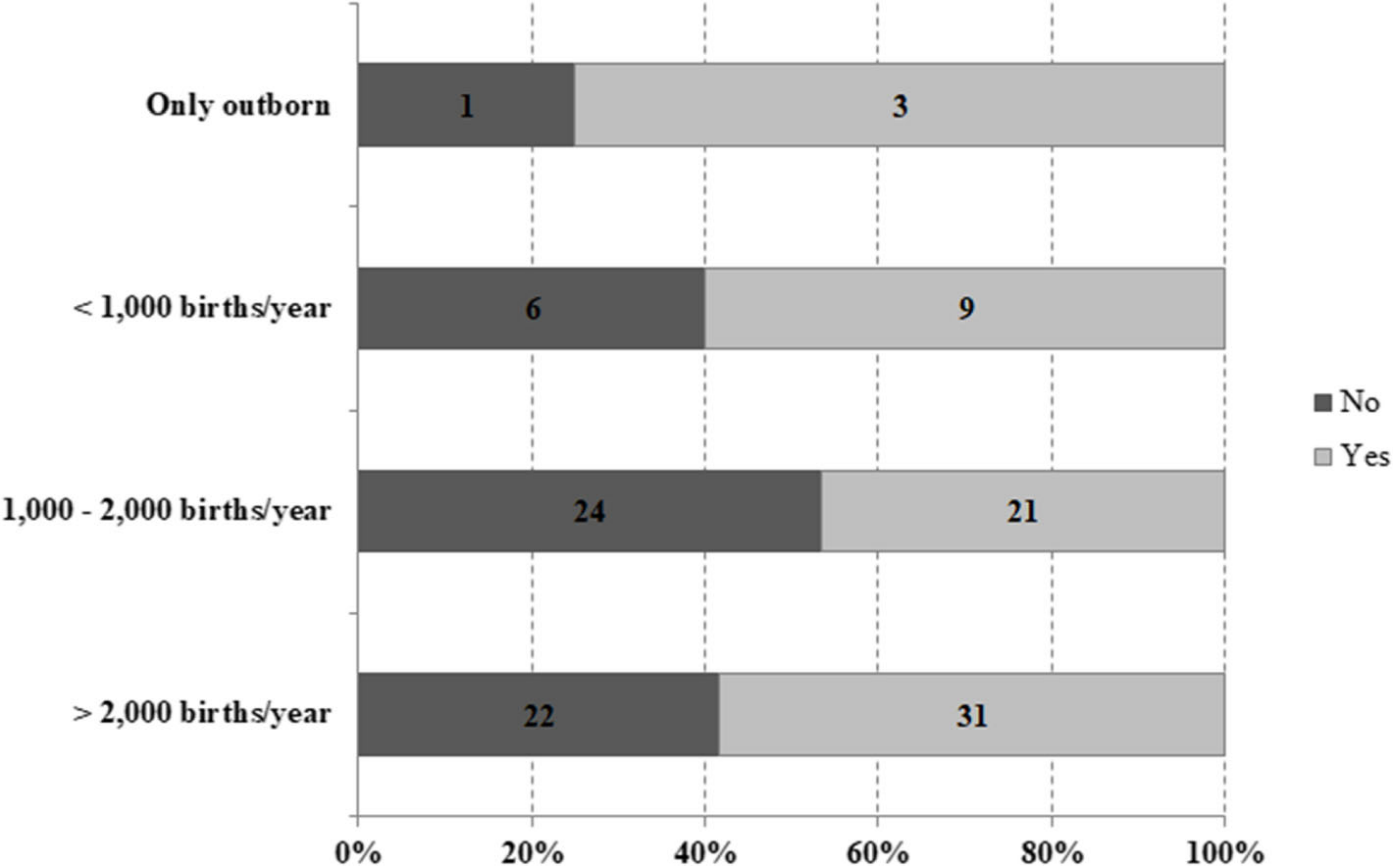
Use of SB for treating MA. Surveyed Italian NICUs (*n* = 117) are classified according to unit’s characteristics. Differences among groups are not significant (*p* value: 0.51)

More than half of the surveyed NICUs (54.7%; 64/117) answered that in their unit it is a common practice to correct MA with intravenous SB. No significant differences were found among centers with different characteristics (*p* value = 0.51), as shown in Fig. 1.

Regarding whether a pH threshold was considered upon deciding to correct MA with SB, 51.6% of centers answered that they treat at pH values < 7.2 (32/62) and 33.9% at pH < 7.1 (21/62). Concerning Base Excess (BE) value thresholds, 69.8% of NICUs (44/63) answered to consider BE values ≤ − 10 and 17.5% (44/63) BE values ≤ − 8 to treat MA with intravenous SB. Lactate values instead were not taken into account for the treatment on behalf of 75.8% of centers (47/62). Results about blood gas analyses criteria used for MA correction with SB are presented in Table 1.

**Table 1:** Criteria for treating MA with SB

***pH***		***Base Excess***		***Lactate***	
	n *(%)*		n *(%)*		n *(%)*
**≤7.10**	21 *(33.9)*	**≤ − 10**	44 *(69.8)*	**no thresholds**	47 (*75.8)*
**≤7.20**	32 *(51.6)*	**≤ − 8**	11 *(17.5)*	**> 5 mmol/l**	13 *(21.0)*
**no thresholds**	5 *(8.1)*	**no thresholds**	5 *(7.9)*	**> 2 mmol/l**	2 *(3.2)*
**≤7.30**	3 *(4.8)*	**≤ − 5**	2 *(3.2)*		
**other**	1 *(1.6)*	**other**	1 *(1.6)*		
Total answers	62 *(100)*		63 *(100)*		62 *(100)*

Blood gas analysis thresholds adopted for the correction of MA with SB

### SB dosage, preparation and administration

69.8% of NICUs (44/63) calculate the SB dose to be administered using the formula” *ml of SB = kg* × *BE* × *0.3 / 2″;* 12.7% of NICUs (8/63) instead use the formula “*ml of SB= kg* × *BE* × *0.3 / 3*”; whereas 9.5% of NICUs (6/63) prefer to use a “full dose” (“*ml of SB = kg* × *BE* × *0.3”*)

Before intravenous administration, 90.5% of the units (57/63) dilute SB with distilled water, while 4.3% of units (5/63) use normal saline. Moreover, the majority of NICUs (87.3%; 55/63) adopts a 1:1 dilution ratio, with only eight units using different ratios (1:2 in 6 units, 1:3 in 1 unit, whereas another unit answered “other”).

61.3% of NICUs (38/62) administer SB by means of a slow 30–60 min intravenous infusion; 27.4% (17/62) prefer to provide SB in “pushes” within 30 min; 8.1% (5/62) use both “pushes” and “slow infusions”, while only 2 units (3.2%) administer SB in 3 to 4 h long infusions

Finally, cases of lesions due to SB extravasations were reported by 8.5% (10/117) of units.

SB dose, preparation and administration practices adopted among Italian NICUs are summarized in Table 2.

**Table 2:** SB dose, preparation and administration practices

***Dose***		***Diluent***		***Dilution***		***Administration time***	
	n *(%)*		n *(%)*		n *(%)*		n *(%)*
***ml of SB = kg*** × ***BE*** × ***0.3 / 2***	44 *(69.8)*	***sterile water for injection***	57 *(90.5)*	**1:1**	55 (*87.3)*	***30–60 min***	38 *(61.3)*
***ml of SB = kg*** × ***BE*** × ***0.3 / 3***	8 *(12.7)*	***normal saline***	5 *(7.9)*	**1:2**	6 *(9.5)*	***≤30 min***	17 (*27.4)*
***ml of SB = kg*** × ***BE*** × ***0.3***	6 *(9.5)*	***other***	1 *(1.6)*	**1:3**	1 *(1.6)*	***bolus + slow infusion***	5 *(8.1)*
***1 mEq/kg***	4 (6.3)			***other***	1 *(1.6)*	***3–4 h or more***	2 *(3.2)*
Total answers	63 *(100)*		63 *(100)*		63 *(100)*		62 *(100)*

### Therapeutic strategies for treating MA

**Fig. 2:**
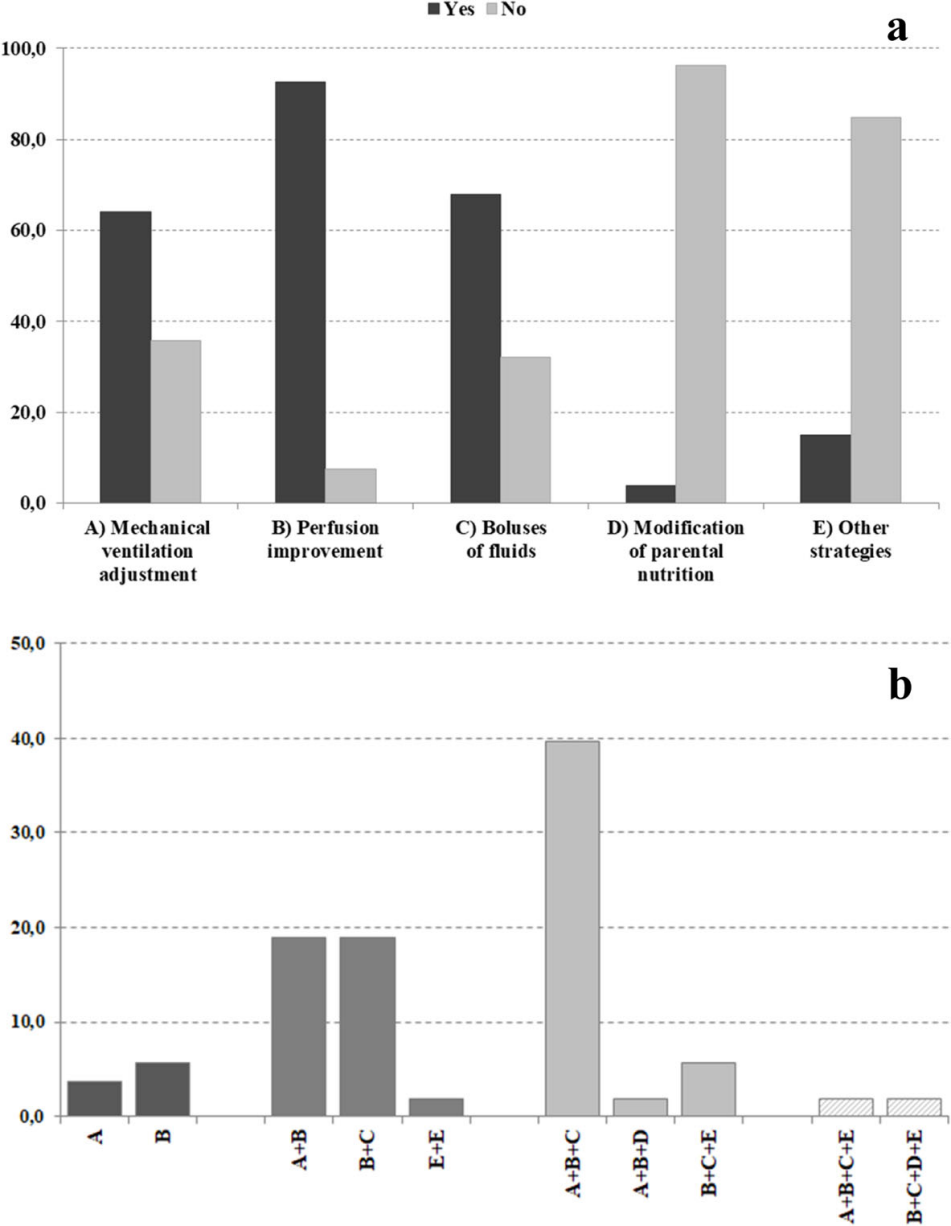
Therapeutic strategies adopted for treating MA in the NICUs not using SB. **a** Percentage of NICUs (*n* = 53) using each strategy. **b** Percentage of NICUs (*n* = 53) adopting one or more therapeutic strategies

All the 53 NICUs, where the treatment of MA with SB is not a common practice, use one or more therapeutic strategies to correct MA (Fig. 2a-b). Ninety-two percent of units (49/53) answered that they turn to perfusion improvement; 68% (36/53) increase the circulating volume with boluses of fluids; 64% (34/53) adjust mechanical ventilation parameters; 4% (2/53) modify parenteral nutrition composition.

### Buffer salts in PN

**Fig. 3:**
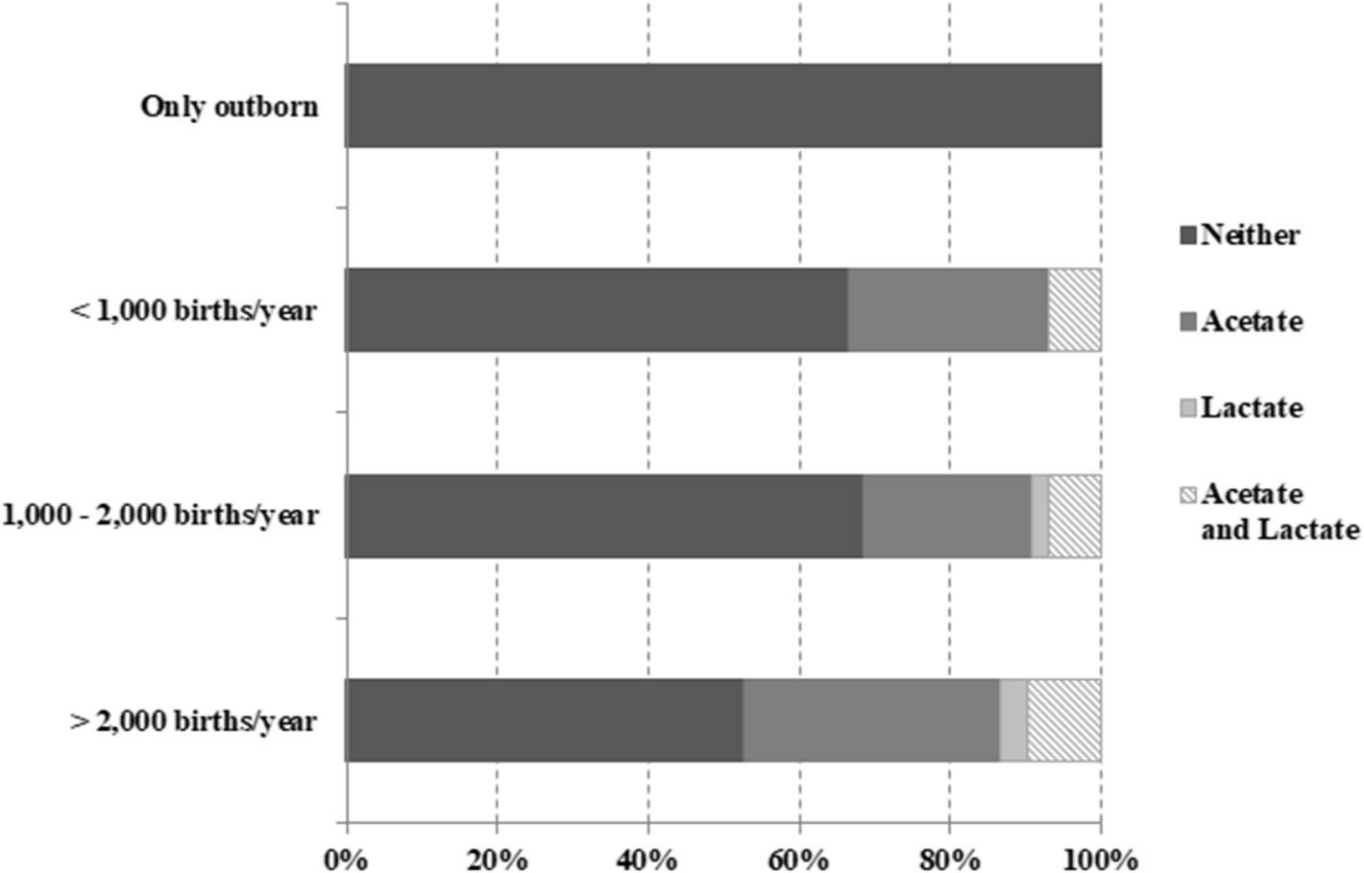
Buffer salts in PN. Percentage of NICUs (*n* = 117) using buffer salts in PN classified according to unit’s characteristics. Differences among groups are not significant (*p* value: 0.76)

62.4% of the participating NICUs (73/117) do not provide buffer systems in PN. On the contrary, 27.4% of centers (32/117) add acetate to the PN, three units (2.6%) adopt lactate, while 9 NICUs (7.7%) administer both lactate and acetate. There is a trend toward a greater use of buffer salts in the units with higher levels of activity, however the difference is not significant (*p* value = 0.76), Fig. 3.

### Use of intravenous SB for other conditions

Metabolic acidosis represents the final result of several illnesses capable of determining an imbalance of acid-base equilibrium. In our research, 56 % of surveyed NICUs (66/117) provide intravenous SB even to treat some conditions not immediately related to MA. The most common scenarios are represented by pathologies with bicarbonate losses (53.8%; 63/117 units) and tubular acidosis (32.5%; 38/117 units).

There were no significant differences in the responses among units with different levels of activity (< 1000 births/year, 1000–2000 births/year, > 2000 births/year, and only outborn). However, the heterogeneity in sample size of the four analyzed groups can be noted.

## Discussion

MA is an acid-base imbalance that can occur acutely or chronically, characterized by a primary reduction in the serum concentration of bicarbonate (HCO3^−^), a secondary decrease in the arterial partial pressure of carbon dioxide (PaCO_2_), and a reduction in blood pH [7]_._ MA is a common finding in the critically ill infant and can be determined by a number of different etiologies, other than renal and gastrointestinal bicarbonate losses (in which the deficit correction with SB has been advocated by several authors [2, 3]). The results of our survey show that more than half of Italian NICUs (54.7%) routinely use SB to treat MA. In the above-mentioned European survey, 42.2% of interviewed neonatologists would have administered SB in the case of an asphyxiated term neonate with severe combined metabolic and respiratory acidosis, with remarkable differences in practices among European countries (the rate of SB use in Italy corresponded to about 40%) [6].

The practice of treating neonatal MA with intravenous SB, was first reported in 1963 by Usher [8], who showed that an early infusion of a 10% glucose solution plus 5–15 mEq/dl of SB resulted in a considerable reduction in mortality among treated neonates. From that moment, this type of infusion, defined as the “Usher regimen”, started to be widely adopted in neonatal care. Nonetheless, in the following years, further researches demonstrated that the administration of SB in asphyxiated neonates was not affecting the acid–base balance in the first 24 h [9], and that there was insufficient evidence from randomized controlled trials to support or refute the use of SB during resuscitation of infants at birth [10]. Furthermore, studies conducted on adult patients evidenced that administration of SB during cardio-pulmonary resuscitation could be detrimental to the myocardial function, due to the worsening of intracellular acidosis related to carbon dioxide accumulation, increased hyperosmolality, extracellular alkalosis and reduced coronary perfusion pressure [3, 11]. Thus, since the 2000 update, the International guidelines for neonatal resuscitation did not suggest to use SB during neonatal resuscitation any longer [12]. However, one shall also mention that other authors are still permissive towards SB therapy [13].

Further researches also showed how the administration of SB could increase the risk of death and intraventricular hemorrhage in preterm infants [4, 5, 14]. In a recent retrospective study, Katheria et al. showed how SB administration in extremely preterm infants does not act on the cardiac output in the short term, but leads to transient fluctuations in cerebral and cardiovascular hemodynamics that could cause dangerous effects on the weak brain vessels of such patients [15]. An increased concentration of CO_2_ (a potent cerebral vasodilator) and greater blood osmolality (resulting in a flow of intracellular water into the extracellular space) have been suggested as possible co-factors for the cerebral blood flow modifications following SB infusion [16].

Our survey evidences the lack of well-defined pH, BE and lactate thresholds adopted by Italian NICUs for the correction of MA with SB.

Such variability could also be explained by means of different criteria used to define neonatal MA in studies thereof [17–19]. Moreover, normal arterial lactate values are influenced by the hours of life of the infant, with described thresholds ranging from more than 3.8 mmol/l (at 48 h.) to over 1.5 mmol/l (after 96 h.) [20].

Around 70% of units that commonly treat MA, when needed, administer “half dose” of the SB amount determined by the classic formula proposed in 1960 by Astrup to correct the BE deficit [21]. In addition, the heterogeneity of SB dilution and administration practices observed reflects the largely arbitrary recommendations that can be found in literature [3, 4, 17, 22].

Intravenous SB solutions are highly hypertonic. The 1:1 dilution is the most used by the surveyed centers and frequently recommended [17–22], yet still being hypertonic.

Evidence is conflicting even with reference to the suggested duration of SB infusions. A systematic review in 2002 reported no differences when comparing rapid vs. slow or no correction [23], whereas, a few years later, other researchers suggested a slow infusion of SB, over a 30-min period, to minimize fluctuations in cerebral blood flow of preterm infants [24].

Our survey showed that there is a relevant number of centers (45%) opting for other strategies for the management of MA, rather than the routine administration of SB. Such approach may be considered as being appropriate, also taking into account that the treatment of neonatal MA should preferably rely on the correction of its primary cause, as also suggested by many experts [1, 3, 6, 11]. Another strategy to deal with chronic MA of preterm infants could be to add buffer salt, such as potassium lactate or sodium acetate, to the PN [25] - a practice reported as being used by 37.6% of the surveyed NICUs.

Preterm infants can be frequently exposed to an excessive chloride intake [26] with subsequent hyperchloremia that can constitute a cause of MA, particularly in premature infants, because of the negative effect on the neonatal kidney capability of eliminating acid load [27]. Thus, limiting chloride infusion and providing sodium and potassium as organic phosphate, sodium acetate-citrate or potassium acetate-citrate within the PN preparation might prevent hyperchloremic metabolic acidosis [28–30]. Moreover, some authors reported as earlier/higher parenteral aminoacid and lipid intakes raised the risk of metabolic acidosis, particularly in babies born less than 24–26 weeks of gestation. Nevertheless, stronger evidence is still needed to further support the mentioned practices.

However, our study includes some limitations. Questions did not distinguish between acute or chronic MA nor between occurrence of such condition in term or preterm infants. Thus, some answers may have been arbitrary and may not have adequately described different strategies adopted. The question regarding the “use of SB only for some pathologies” was not fully clear, and we have received answers both from the units that were using SB as well as those not routinely using it. Finally, answers given by the directors of the NICUs did not always reflect attitudes of every clinician within the unit.

## Conclusions

The conducted survey highlighted how, in Italian NICUs, SB is a commonly adopted treatment for MA and that prescription criteria, dosage and time of infusion vary widely across centers. According to scientific evidence, management of neonatal MA should be directed towards the diagnosis and solution of the possible underlying disorders, reserving the use of SB only for selected cases, also considering the severity of its adverse effects and the lack of efficacy-based evidence. It is envisaged that a renewed focus and guidance on behalf of scientific societies on the suitable management of MA would likely contribute to harmonizing the current heterogeneity of practices, reducing the use of potentially unsafe treatments.

## Supplementary Information

**Additional file 1.**

**Additional file 2.**

**Additional file 3.**

## Data Availability

The datasets used and analysed during the current study data are available from the Neonatal Pharmacotherapy Study Group of the Italian Society of Neonatology (SIN) on reasonable request and with permission of the Italian Society of Neonatology board.

## Abbreviations

BE: Base excess
SIN: Italian society of neonatology
MA: Metabolic acidosis
NICU: Neonatal intensive care unit
PN: Parenteral nutrition
SB: Sodium bicarbonate
SD: Standard deviation
