# Supplementary material for: Use of intravenous sodium bicarbonate in neonatal intensive care units in Italy: a nationwide survey
Source: Ital J Pediatr. 2021 Mar 11;47:63. doi: 10.1186/s13052-021-00955-3 (PMC7953611; doi:10.1186/s13052-021-00955-3)
Supplement: Supplementary file 1 — Additional file 1. [file 13052_2021_955_MOESM1_ESM.pdf]

Utilizzo del Bicarbonato di Sodio nelle Unità di Terapia Intensiva Neonatale in Italia

Caro Collega,
come GdS di Farmacoterapia Neonatale, stiamo conducendo una indagine conoscitiva sull'utilizzo del bicarbonato di sodio in Terapia Intensiva Neonatale (UTIN) in Italia. La invitiamo a completare questo breve questionario, che abbiamo inviato a tutti i direttori delle UTIN presenti sul territorio nazionale. I dati raccolti verranno trattati in forma anonima e confidenziale. I risultati definitivi verranno presi in considerazione per una eventuale pubblicazione su riviste scientifiche *peer-reviewed*.
Non esiti a contattarci per ulteriori chiarimenti:

email: [redacted] mobile: +39 [redacted]
email: [redacted] mobile: +39 [redacted]

Grazie in anticipo per il tempo e la collaborazione.
GdS Farmacoterapia Neonatale

Può rispondere al questionario online all'indirizzo
https://goo.gl/forms/nMn3KTyNb6xNQv9K2
oppure inviare una scansione/foto del questionario compilato via email o mediante Whatsapp ai riferimenti sopra citati

1) **La UTIN che Lei dirige annualmente assiste:**
   □ <1000 nati □ 1000-2000 nati □ >2000 nati □ solo neonati outborn

2) **Nella UTIN che Lei dirige è pratica comune la correzione dell'acidosi metabolica con $NaHCO_3$ e.v.?:**
   □ no □ si

3) **Se ha risposto "No" alla domanda n. 2), quali altre strategie terapeutiche vengono impiegate per la correzione dell'acidosi metabolica?** (anche più di una risposta)**:**
   □ modifica dei parametri ventilatori
   □ miglioramento della perfusione
   □ boli di volume
   □ altro (specificare) ……………………………………………………………………

4) **Se ha risposto "Si" alla domanda n. 2), avete dei valori limite di pH per la correzione con $NaHCO_3$ ev?**
   □ no
   □ pH ≤ 7,30 □ pH ≤ 7,20 □ pH ≤ 7,10
   □ altro (specificare) ……………………………………………………………………

5) **Se ha risposto "Si" alla domanda n. 2), avete dei valori limite di EB per la correzione con $NaHCO_3$ ev?**
   □ no
   □ EB ≤ -5 mEq/L □ EB ≤ -8 mEq/L □ EB ≤ -10 mEq/L
   □ altro (specificare) ……………………………………………………………………

6) **Se ha risposto "Si" alla domanda n. 2), avete dei valori limite di Lattati per la correzione con $NaHCO_3$ ev?**
   □ no
   □ Lattati >2mmol/L □ Lattati >5mmol/L

□ altro (specificare) ………………………………………………………………………….

7) **Se hai risposto "Si" alla domanda n. 2), che dosaggio di $NaHCO_3$ utilizzate rispetto alla formula per la correzione del deficit (peso x EB x 0,3)?**
□ la dose totale
□ metà dose
□ un terzo della dose
□ 1mEq/kg
□ altro (specificare) ………………………………………………………………………….

8) **Se hai risposto "Si" alla domanda n. 2), cosa utilizzate per la diluizione?**
□ acqua distillata
□ soluzione fisiologica
□ altro (specificare) ………………………………………………………………………….

9) **Se hai risposto "Si" alla domanda n. 2), che rapporto utilizzate per la diluizione?**
□ 1:1
□ 1:2
□ 1:3
□ 1:4
□ altro (specificare) ………………………………………………………………………….

10) **Se hai risposto "Si" alla domanda n. 2), che modalità di somministrazione utilizzate?**
□ bolo ev (≤30 min)
□ infusione lenta (30-60 min)
□ bolo + infusione lenta
□ altro (specificare) ………………………………………………………………………….

11) **Nella UTIN che Lei dirige utilizzate il $NaHCO_3$ solo in alcune particolari patologie?**
□ no   □ si

12) **Se nella UTIN che Lei dirige utilizzate il $NaHCO_3$ solo in alcune patologie, quali sono?**
(anche più di una risposta)
□ acidosi tubulare
□ patologie con perdita di bicarbonato
□ altro (specificare) ………………………………………………………………………….

13) **Nei componenti della Nutrizione Parenterale prescritta nella UTIN che lei dirige sono presenti …**
□ acetato
□ lattato
□ acetato e lattato
□ non sono presenti acetato e lattato
□ altri sistemi tampone (specificare)………………………………………………………

14) **Nella UTIN che Lei dirige si sono mai verificate lesione da stravaso di $NaHCO_3$?**
□ no   □ si

Commenti al questionario

....
....
....
....
