# Supplementary material for: Use of intravenous sodium bicarbonate in neonatal intensive care units in Italy: a nationwide survey
Source: Ital J Pediatr. 2021 Mar 11;47:63. doi: 10.1186/s13052-021-00955-3 (PMC7953611; doi:10.1186/s13052-021-00955-3)
Supplement: Supplementary file 2 — Additional file 2. [file 13052_2021_955_MOESM2_ESM.pdf]

List of contacted Neonatal Intensive Care Units (n = 120). 117 units participated in the survey, 3 units did not participate in the survey.

Neonatal Intensive Care Unit	City	Region
Università G. d'Annunzio	Chieti	Abruzzo
Presidio Ospedaliero S. Salvatore	L'Aquila	Abruzzo
Ospedale Civile dello Spirito Santo	Pescara	Abruzzo
Azienda Ospedaliera San Carlo	Potenza	Basilicata
Azienda Ospedaliera "Pugliese - Ciaccio"	Catanzaro	Calabria
Azienda Ospedaliera Cosenza	Cosenza	Calabria
Ospedale Civile San Giovanni di Dio	Crotone	Calabria
Azienda Ospedaliera Reggio Calabria	Reggio Calabria	Calabria
Ospedale S. Giuseppe Moscati	Avellino	Campania
Ospedale S. Maria Della Speranza	Battipaglia	Campania
Ospedale Rummo	Benevento	Campania
Ospedale Sacro Cuore - Fatebenefratelli	Benevento	Campania
Azienda Ospedaliera di Caserta	Caserta	Campania
PO "Pineta Grande"	Castel Volturno	Campania
Azienda Ospedaliera Monaldi	Napoli	Campania
Fondazione Evengelica Betania - Ospedale Evangelico di Napoli	Napoli	Campania
Azienda Universitaria Policlinico Federico II	Napoli	Campania
Ospedale Buon Consiglio - Fatebenefratelli	Napoli	Campania
Ospedale Santissima Annunziata	Napoli	Campania
A.O.R.N. Santobono Pausilipon	Napoli	Campania
Nocera Inferiore - Pagani	Nocera Inferiore	Campania
AOU OO. RR. S. Giovanni di Dio e Ruggi d'Aragona	Salerno	Campania
Ospedale Maggiore	Bologna	Emilia Romagna
Azienda Ospedaliera Policlinico S. Orsola - Malpighi	Bologna	Emilia Romagna
Ospedale Bufalini	Cesena	Emilia Romagna
Azienda Ospedaliera Universitaria S. Anna	Ferrara	Emilia Romagna
Azienda Ospedaliera Policlinico	Modena	Emilia Romagna
Azienda Ospedaliera Universitaria	Parma	Emilia Romagna
Ospedale di Ravenna	Ravenna	Emilia Romagna
Ospedale Santa Maria Nuova	Reggio Emilia	Emilia Romagna
Ospedale Infermi	Rimini	Emilia Romagna
Ospedale Santa Maria degli Angeli di Pordenone	Pordenone	Friuli Venezia Giulia
IRCCS Burlo Garofolo	Trieste	Friuli Venezia Giulia
Ospedale Santa Maria della Misericordia	Udine	Friuli Venezia Giulia
AO San Camillo - Forlanini	Roma	Lazio
Policlinico Universitario Umberto I	Roma	Lazio
Azienda Ospedaliera San Giovanni Addolorata	Roma	Lazio
Ospedale Pediatrico Bambino Gesù	Roma	Lazio
Ospedale Sant'Eugenio	Roma	Lazio
Ospedale Fatebenefratelli San Pietro	Roma	Lazio
Ospedale Fatebenefratelli San Giovanni Calibita - Isola Tiberina	Roma	Lazio
Policlinico Casilino	Roma	Lazio
Fondazione Policlinico Agostino Gemelli	Roma	Lazio
Ospedale Belcolle	Viterbo	Lazio
Ospedale San Martino	Genova	Liguria
Istituto Giannina Gaslini	Genova	Liguria
ASST Papa Giovanni XXIII	Bergamo	Lombardia
ASST Spedali Civili	Brescia	Lombardia

Fondazione Poliambulanza Istituto Ospedaliero	Brescia	Lombardia
Ospedale Valduce	Como	Lombardia
Ospedale Alessandro Manzoni	Lecco	Lombardia
Ospedale Maggiore	Lodi	Lombardia
Ospedale Poma	Mantova	Lombardia
ASST Fatebenefratelli Sacco Ospedale Macedonio Melloni	Milano	Lombardia
ASST Fatebenefratelli Sacco Ospedale dei Bambini Vittore Buzzi	Milano	Lombardia
ASST Grande Ospedale Metropolitano Niguarda	Milano	Lombardia
Fondazione IRCCS Ca' Granda Ospedale Maggiore Policlinico	Milano	Lombardia
Azienda Ospedaliera San Gerardo	Monza	Lombardia
Fondazione IRCCS Policlinico San Matteo	Pavia	Lombardia
Ospedale di Rho	Rho	Lombardia
ASST Lariana Ospedale Sant’Anna	San Fermo della Battaglia	Lombardia
Azienda Ospedaliera Bolognini Seriate	Seriate	Lombardia
Ospedale F. Del Ponte	Varese	Lombardia
Ospedale Pediatrico Salesi	Ancona	Marche
Ospedale A. Cardarelli	Campobasso	Molise
Azienda Ospedaliera Nazionale SS. Antonio e Biagio e Cesare Arrigo	Alessandria	Piemonte
Azienda Sanitaria Ospedaliera S. Croce e Carle	Cuneo	Piemonte
ASL TO5 Ospedale Santa Croce	Moncalieri	Piemonte
Azienda Ospedaliera Maggiore della Carità	Novara	Piemonte
AO Ordine Mauriziano Ospedale Umberto I	Torino	Piemonte
PO Maria Vittoria	Torino	Piemonte
Ospedale Infantile Regina Margherita	Torino	Piemonte
Ospedale Generale Regionale “F. Miulli”	Acquaviva delle Fonti	Puglia
Ospedale “Di Venere” - Carbonara	Bari	Puglia
AOU Consorziale Policlinico	Bari	Puglia
Azienda Ospedaliera "A. Perrino"	Brindisi	Puglia
Ospedali Riuniti	Foggia	Puglia
Azienda Ospedaliera "Vito Fazzi"	Lecce	Puglia
Ospedale Casa Sollievo della Sofferenza	San Giovanni Rotondo	Puglia
Azienda Ospedaliera SS. Annunziata di Taranto	Taranto	Puglia
Azienda Ospedaliera "Card. G. Panico" di Tricase	Tricase	Puglia
Azienda Ospedaliera Universitaria Clinica Pediatrica Macciotta	Cagliari	Sardegna
Azienda Ospedaliera Universitaria di Sassari	Sassari	Sardegna
ASP Agrigento POS S. Giovanni di Dio	Agrigento	Sicilia
ARNAS Garibaldi	Catania	Sicilia
“AOU Policlinico – Vittorio Emanuele” PO Santo Bambino	Catania	Sicilia
ARNAS Garibaldi	Catania	Sicilia
AO Cannizzaro	Catania	Sicilia
“AOU Policlinico – Vittorio Emanuele” PO Gaspare Rodolico	Catania	Sicilia
ASP Enna PO Umberto I	Enna	Sicilia
AOUP Messina	Messina	Sicilia
Azienda Ospedaliera Papardo	Messina	Sicilia
ARNAS Civico	Palermo	Sicilia
Ospedale Buccheri La Ferla Fatebenefratelli	Palermo	Sicilia
ASL Palermo PO Ingrassia	Palermo	Sicilia
Ospedali Riuniti Cervello -Villa Sofia	Palermo	Sicilia
Università di Palermo - Azienda Policlinico	Palermo	Sicilia
ASP Messina PO Patti	Patti	Sicilia
ASP Ragusa PO Maria Paterno' Arezzo	Ragusa	Sicilia
ASP Siracusa PO Umberto I	Siracusa	Sicilia

ASP Messina PO Taormina	Taormina	Sicilia
ASP Trapani POS Antonio Abate	Trapani	Sicilia
Ospedale San Donato	Arezzo	Toscana
AOU Careggi	Firenze	Toscana
AOU Meyer	Firenze	Toscana
Nuovo Ospedale San Giovanni di Dio	Firenze	Toscana
Ospedale Versilia - Lido di Camaiore	Lido di Camaiore	Toscana
AOU Pisana - Stabilimento di Santa Chiara	Pisa	Toscana
Ospedale di Prato Santo Stefano	Prato	Toscana
Azienda Ospedaliera Universitaria Senese - Ospedale Santa Maria alle Scotte	Siena	Toscana
Ospedale di Bolzano	Bolzano	Trentino-Alto Adige
Ospedale S.Chiara	Trento	Trentino-Alto Adige
Azienda Ospedaliera di Perugia	Perugia	Umbria
Azienda Ospedaliera Santa Maria	Terni	Umbria
Ospedale di Camposampiero	Camposampiero	Veneto
Ospedale dell'Angelo	Mestre	Veneto
Azienda Ospedaliera Policlinico di Padova	Padova	Veneto
Ospedale Ca' Foncello	Treviso	Veneto
Azienda Ospedaliera Universitaria Integrata di Verona	Verona	Veneto
Ospedale San Bortolo	Vicenza	Veneto
