# Supplementary material for: Use of intravenous sodium bicarbonate in neonatal intensive care units in Italy: a nationwide survey
Source: Ital J Pediatr. 2021 Mar 11;47:63. doi: 10.1186/s13052-021-00955-3 (PMC7953611; doi:10.1186/s13052-021-00955-3)
Supplement: Supplementary file 3 — Additional file 3. [file 13052_2021_955_MOESM3_ESM.doc]

**Use of Sodium Bicarbonate in Neonatal Intensive Care Units in Italy**

Dear Colleague,

as a study group of Neonatal Pharmacotherapy, we are conducting a survey on the use of sodium bicarbonate in the Neonatal Intensive Care Units (NICU) in Italy. We invite you to complete this short questionnaire that we sent to all the directors of the NICUs residing in the national territory. The data collected will be treated anonymously and confidentially. The final results will be taken into consideration for a possible publication in peer-reviewed scientific journals.

Do not hesitate to contact us for further clarification:

email: …………………………… mobile: ……………………

email: …………………………… mobile: ……………………

Thanks in advance for your time and cooperation.

The Neonatal Pharmacotherapy Study Group

You can answer the online questionnaire at

<https://goo.gl/forms/nMn3KTyNb6xNQv9K2>

or send a scan / photo of the completed questionnaire via email or via WhatsApp to the references mentioned above

1. **1) The NICU that you direct, annually take care for:**

□ <1000 neonates □ 1000-2000 neonates □ >2000 neonates □ only outborn neonates

1. **In the NICU that you direct, it is a common practice to correct metabolic acidosis with i.v. NaHCO_3_ ?:**

□ no □ yes

1. **If you have answered “no” to the question n. 2, what other therapeutic strategies are used for the correction of metabolic acidosis?** (multiple responses are allowed)**:**

□ adjustment of mechanical ventilation parameters

□ perfusion improvement

□ boluses of fluids

□ other (please specify) ………………………………………………………………….

1. **If you have answered “yes” to the question n. 2, do you have pH limit values for the correction with i.v. NaHCO_3_?**

□ no

□ pH ≤ 7.30 □ pH ≤ 7.20 □ pH ≤ 7.10

□ other (please specify) .………………………………………………………………….

1. **If you have answered “yes” to the question n. 2, do you have BE limit values for the correction with i.v. NaHCO_3_?**

□ no

□ BE ≤ -5 mEq/L □ BE ≤ -8 mEq/L □ BE ≤ -10 mEq/L

□ other (please specify) ..………………………………………………………………….

1. **If you have answered “yes” to the question n. 2, do you have Lactate limit values for the correction with i.v. NaHCO_3_?**

□ no

□ Lactate >2mmol/L □ Lactate >5mmol/L

□ other (please specify) …………………………………………………………………..

1. **If you have answered “yes” to the question n. 2, which NaHCO_3_ dose do you use, compared to the formula for correction of the deficit (weight x BE x 0.3)?**

□ the full dose

□ half dose

□ one third of the dose

□ 1mEq/kg

□ other (please specify) …………………………………………………………………

1. **If you have answered “yes” to the question n. 2, which do you use for dilution?**

□ distilled water

□ normal saline

□ other (please specify) …………………………………………………………………..

1. **If you have answered “yes” to the question n. 2, which dilution ratio do you use?**

□ 1:1

□ 1:2

□ 1:3

□ 1:4

□ other (please specify) …………………………………………………………………….

1. **If you have answered “yes” to the question n. 2, which administration rate do you use?**

□ i.v. bolus (≤30 min)

□ slow infusion (30-60 min)

□ bolus + slow infusion

□ other (please specify) …………………………………………………………………….

1. **In the NICU that you direct, do you use NaHCO_3_ only for some particular pathologies?**

□ no □ yes

1. **If, in the NICU that you direct, you do use NaHCO_3_ only for some pathologies, which are they?** (multiple responses are allowed)

□ tubular acidosis

□ pathologies with loss of bicarbonate

□ other (please specify) …………………………………………………………………….

1. **In the components of Parenteral Nutrition prescribed in the NICU that you direct are present ...**

□ acetate

□ lactate

□ acetate and lactate
□ there are no acetate and lactate

□ other buffer systems (please specify) ……………………………………………………….

1. **In the NICU that you direct, have any NaHCO_3_ extravasation injury ever occurred?**

□ no □ yes

**Comments to the questionnaire**

…………………………………………………………………………………………

…………………………………………………………………………………………

…………………………………………………………………………………………

…………………………………………………………………………………………
